# Sequential Pontine Strokes With the “Heart Appearance” Sign

**DOI:** 10.7759/cureus.15025

**Published:** 2021-05-14

**Authors:** Carlos Eduardo A Mantese, Mariana Pontalti, Alexandre Guerreiro, Charles Klamt

**Affiliations:** 1 Neurology, Hospital Mãe de Deus, Porto Alegre, BRA

**Keywords:** stroke, pontine stroke, sequential stroke, heart appearance, branch artery disease

## Abstract

Most brainstem infarcts are caused by pontine strokes. When a bilateral pontine or brainstem stroke occurs, it can result in an image called the “heart appearance” sign. Sequential pontine strokes have not yet been described to cause the “heart appearance” sign. We report a case of sequential pontine strokes in a 47-year-old man with no relevant medical history. He presented with acute left-sided hemiparesis. Initial brain MRI showed a right-sided pontine infarction. Two weeks later, the patient’s left side hemiparesis and dysarthria worsened, and he developed right-sided facial paralysis. The brain MRI showed a left-sided pontine infarction that looked like the “heart appearance” sign. The patient had a good recovery. This report highlights a case of atypical pontine stroke recurrence two weeks apart and discusses branch artery disease as a possible etiologic cause.

## Introduction

Pontine stroke accounts for most brainstem infarcts [1]. When a bilateral pontine or brainstem stroke occurs, it can result in an image called the “heart appearance” sign [2]. Most pontine strokes are unilateral; however, approximately 10-33% of patients exhibit a bilateral acute pontine infarct [1,3]. Approximately 25% of patients have a progressive course, usually within four days of the first stroke [4]. Herein, we report a case of unilateral pontine stroke with a contralateral recurrence two weeks after the first stroke, in which the “heart appearance” sign was visible.

## Case presentation

**Figure 1:**
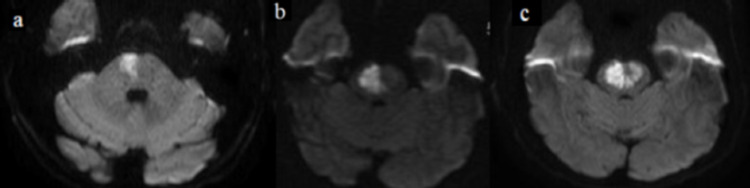
MRI images. a. Diffusion-weighted increased signal on the right pontine side. b. Right-sided signal expansion. c. New diffusion-weighted increased signal on the left side, similar to the “heart appearance” sign.

A 47-year-old male patient with no relevant medical history presented with left hemiparesis for one hour. He had minor left facial paralysis and dysarthria, severe hemiparesia pointing 9 on the National Institutes of Health Stroke Scale. Brain tomography and angiotomography were performed; no brain lesion, arterial stenosis, or occlusion on cerebral circulation was visible. With clinical history, physical exam and image were compatible with stroke with significant deficits, intravenous thrombolysis was performed. The following day, MRI showed diffusion-weighted increased signal on the right pontine side as expected to a stroke (Figure 1a). A transesophageal echocardiogram was normal. Initial investigation for rheumatologic and hematologic was normal. The patient had a moderate continuous recovery. Five days after admission, he was discharged to ambulatory care follow-up and was prescribed statins and aspirin. At discharge, neurologic examination revealed left-sided hemiparesis and mild dysarthria.

Two weeks after the initial stroke, the patient returned to the ED due to worsening left hemiparesis and dysarthria. MRI showed an expansion of the area of diffusion-weighted increased signal on the right pontine side (Figure 1b); the patient was managed as a stroke expansion. No indications of infection or hypotension were noted. However, the patient developed right central facial paresis and anarthria 12 hours after admission. A more comprehensive evaluation was performed with brain contrast MRI due to the new symptoms and longer time frame between the first stroke and stroke expansion than expected. The new brain MRI revealed a left pontine infarction (Figure 1c) with the characteristic “heart appearance” sign. We performed an extensive search for stroke etiology with negative results: arteriography and lumbar puncture were normal and there was no evidence of thrombophilias. After the initial worsening, the patient stabilized and had a stable recovery. He was discharged one month after the second hospital admission, with left-sided hemiplegia, severe dysarthria and dysphagia, right side ataxia, and a Modified Rankin Scale score of 4. Home recovery was slow, and the ability to walk with no assistance was gradually regained. In a follow-up transesophageal echocardiogram, a patent foramen ovale (PFO) with shunt was found. The patient was referred for PFO closure after several 24-hour Holter monitoring.

## Discussion

The “heart appearance” sign on MRI has been described for bilateral infarctions in different brainstem regions, including the midbrain [2], pons [5], and medulla [6]. In these locations, strokes can be associated with large artery disease; however, reports describing a normal basilar artery also exist [1]. To our knowledge, none of these “heart appearance” images have been described as sequential strokes. Thus, the presentation of this case was intriguing and we had to expand the differential diagnosis. This particular case is unique because two different strokes in the pontine region occurred two weeks apart. Some pontine strokes have a progressive course, usually within the first few days [4]. The image and evolution made this case challenging. This article highlights the unusual presentation of two strokes two weeks apart.

We performed an extensive etiology examination; however, the only abnormal examination was PFO. The strokes were located in the territory of the perforating arteries. We excluded lipohyalinosis from diagnosis because the patient had no relevant risk factors and had an unusual recurrence time. The patient was referred for PFO closure after significant discussion, as he was young and no other etiology was found during examination. 

Another possible cause is branch artery disease. This disease can be located at the atheromatous junction. Branch artery disease can be caused by luminal plaques obstructing a branch, junctional plaques extending into a branch, or microatheroma forming within the orifice of a branch [7]. A previous study on 150 patients with isolated pontine infarcts found only 14 had bilateral pontine infarcts [3]. A few patients had a transient ischemic attack two weeks before the pontine infarct, and six had a progressive course. The bilateral pontine infarct etiology was thought to be due to either basilar artery atheroma causing occlusion of the pontine perforating arteries or small-artery disease. Therefore, despite some patients having a progressive course, it is an acute progression. In 1977, Fisher reported a case of an acute bilateral stroke arising from two pontine branches, one of which supplied a few millimeters of contralateral tissue [8]. Considering our patient had strokes two weeks apart, we do not believe he had only one branch artery occlusion and progression but rather two branch arteries by a luminal or junctional plaque, which were not visible on angiography. Thus, the first stroke destabilized the second branch.

## Conclusions

This case highlights important findings that will assist in the recognition and etiologic examination of pontine strokes. We discussed the case of a patient who had two strokes in the pons two weeks apart. Progression in stroke patients is common; however, recurrence is not. The recurrence in this patient led to the development of the “heart appearance” sign on MRI. We also highlight the possibility that branch artery disease may be the cause.
